# Re-sequencing and morphological data revealed the genetics of stone shell and kernel traits in apricot

**DOI:** 10.3389/fpls.2023.1196754

**Published:** 2023-05-30

**Authors:** Qiuping Zhang, Yuping Zhang, Weisheng Liu, Ning Liu, Xiaoxue Ma, Chunjing Lü, Ming Xu, Shuo Liu, Yujun Zhang

**Affiliations:** Liaoning Institute of Pomology, Yingkou, China

**Keywords:** apricot, kernel traits, stone shell traits, genome-wide association study (GWAS), candidate gene analyses

## Abstract

Kernel-using apricot (*Prunus armeniaca* L.) is an economically important fruit tree species in arid areas owing to its hardiness and cold and drought tolerance. However, little is known about its genetic background and trait inheritances. In the present study, we first evaluated the population structure of 339 apricot accessions and the genetic diversity of kernel-using apricots using whole genome re-sequencing. Second, the phenotypic data of 222 accessions were investigated for two consecutive seasons (2019 and 2020) for 19 traits, including kernel and stone shell traits and the pistil abortion rate of flowers. Heritability and correlation coefficient of traits were also estimated. The stone shell length (94.46%) showed the highest heritability, followed by the length/width ratio (92.01%) and length/thickness ratio (92.00%) of the stone shell, whereas breaking force of the nut (17.08%) exhibited a very low heritability. A genome-wide association study (GWAS) using general linear model and generalized linear mixed model revealed 122 quantitative trait loci (QTLs). The QTLs of the kernel and stone shell traits were unevenly assigned on the eight chromosomes. Out of the 1,614 candidate genes identified in the 13 consistently reliable QTLs found using the two GWAS methods and/or in the two seasons, 1,021 were annotated. The sweet kernel trait was assigned to chromosome 5 of the genome, similar to the almond, and a new locus was also mapped at 17.34–17.51 Mb on chromosome 3, including 20 candidate genes. The loci and genes identified here will be of significant use in molecular breeding efforts, and the candidate genes could play essential roles in exploring the mechanisms of genetic regulation.

## 1 Introduction

Apricots are widely cultivated in the Northern Hemisphere and can be classified as fresh-using, kernel-using, and ornamental apricots according to their utilization purpose. Because of their hardiness and cold and drought tolerance (Zhang et al., 2022a), kernel-using apricots are an economically important fruit tree species in arid areas. In China, the area and annual output of kernel-using apricots are approximately 957,000 hm² and 300,000 t, respectively.

**Figure 1:**
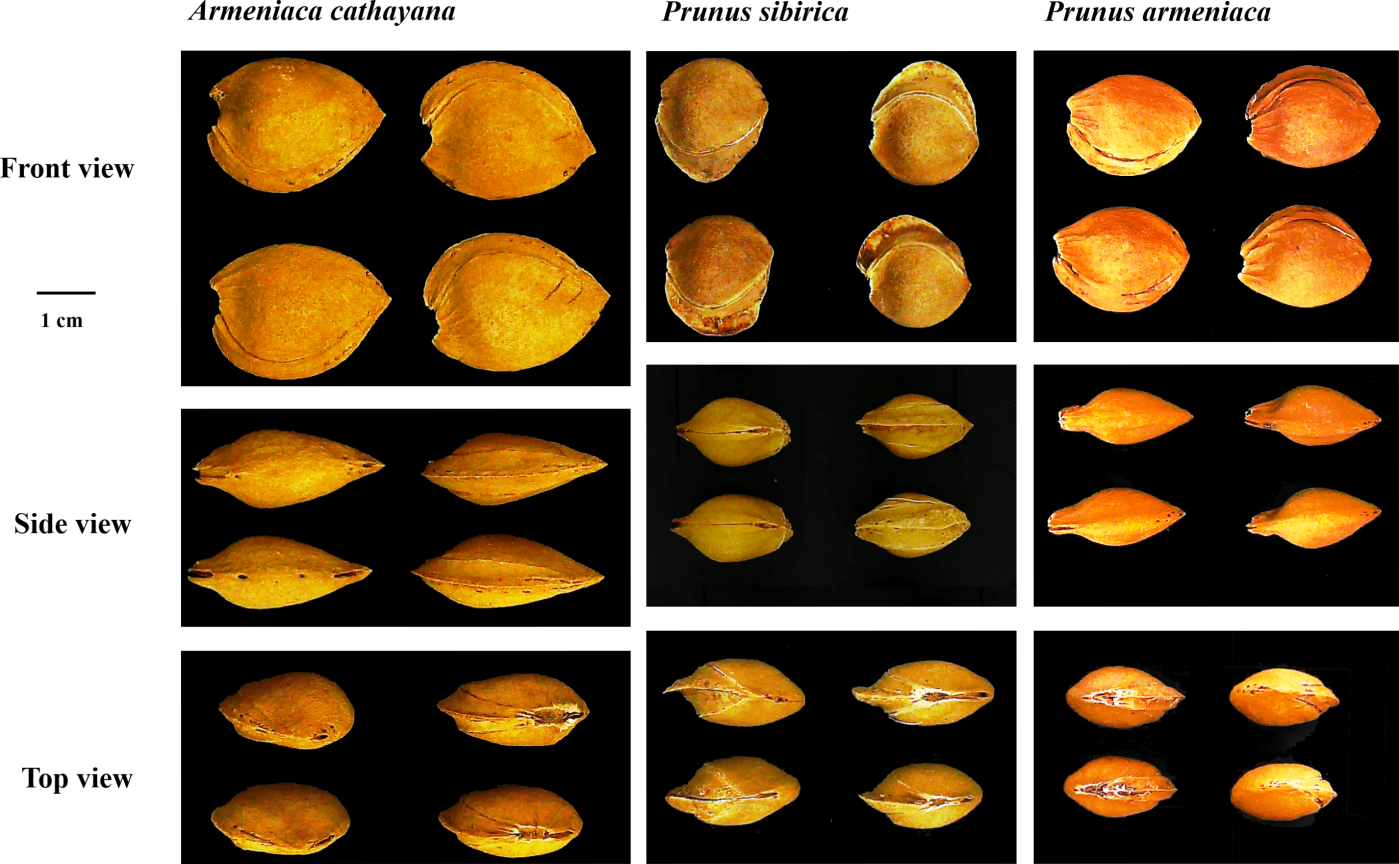
The morphological characteristics of stone in *Armeniaca cathayana* cv. Yiwofeng, *Prunus sibirica* cv. Liaomei, and *Prunus armeniaca* cv. Chuanzhihong.

Cultivated apricots belong to common apricots (*Prunus armeniaca* L.). Despite an undocumented history, the kernel-using apricot is known to have been grown for a long time in north Hebei Province (Zhuolu County) and has an overlapping area with wild populations of *Prunus sibirica* L. Its botanical features appear to be similar to those of *Prunus armeniaca* L. and *P. sibirica*; however, it has some particular traits that can be distinguished from *P. armeniaca* and *P. sibirica* (Liu et al., 2012) (**Figure 1**). Consequently, apricots with large stones and the special traits mentioned above were considered a new species of Rosaceae by Fu et al. (2010) and named *Armeniaca cathayana* D. L. Fu et al. Long before it was named, the view that this type of apricot possibly originated from interspecific hybridization between *P. armeniaca* and *P. sibirica* was also supported based on the peroxidase isozyme (Lü et al., 1994) and was later verified as nuclear genome markers (Zhang et al., 2014; Fu et al., 2016). Based on maternal inheritance markers (chloroplast simple-sequence repeats), *P. armeniaca* was considered the female progenitor of *A. cathayana* (Zhang et al., 2018). Traditional popular kernel-using apricot cultivars include “Longwangmao”,“Yiwangfeng”, and “Baiyubian”, “Guoren”, “Fengren”, and “Youyi” are improved cultivars that have been bred using the clonal selection method. Although a high level of genetic heterozygosity was attributed to two-genome recombination, the population of *A. cathayana* has low diversity (Zhang et al., 2014; Zhang et al., 2021). Therefore, it is crucial to increase the number of new genes and expand the genetic background to breed kernel-using apricots. Few fresh apricot cultivars have been used in the breeding of kernel-using apricots, which may be related to the lack of evaluation of their stone shell and kernel traits.

Breeders of kernel-using apricots have always selected for kernel quality with respect to size, taste, shape, and kernel yield, as well as attempted to improve the crushing performance of stone, shell, and pistil abortions. The efficiency of cross-breeding programs mainly depends on the choice of progenitors and knowledge of the inheritance of traits that are to be improved. However, there are few reports on the genetics of these traits, most of which focus on fruit quality traits (Ruiz et al., 2010; García-Gómez et al., 2019; Zhang et al., 2022b), self-compatibility (Vilanova et al., 2003), and the resistance to *Sarka* virus (Hurtado et al., 2002) in fresh-using apricots. The bitter taste of apricot kernels is caused by the toxic cyanogenic diglucoside amygdalin (Negri et al., 2008). The loss of cyanohydrins from the seed kernels was initially described as monogenic and dominant in apricots (Gomez et al., 1998) and almonds (Dicenta and García, 1993). The locus of sweet kernel (SK) in almond was assigned to chromosome 5 of the genome by map-based cloning, and this locus was shown to be a basic helix-loop-helix transcription factor that is considered responsible for P450 monooxygenase–encoding genes (*PdCYP79D16* and *PdCYP71AN24*) transcription (Sánchez-Pérez et al., 2019). However, the “sweet kernel” was characterized as a recessive trait, controlled by a monogenic single gene in peach, linked to the fuzzless skin trait (Werner and Creller, 1997). Furthermore, Negri et al. (2008) proposed that based on the cyanoglucoside content in apricot, a multi-gene model controlled this trait using five non-linked loci. Therefore, the molecular and genetic regulation of SK requires further investigation.

The inheritance of some stone shell and kernel traits has been reviewed in almonds (Socias i Company et al., 2009; Martínez-García et al., 2019), which is a species related to the kernel-using apricots. In almond, the kernel size and weight measurements exhibited high heritability values (0.62 to 0.81), whereas retention of outer shell (0.34) and width of opening (0.21) showed lower heritability (Kester et al., 1977). Arteaga and Socias i Company (2001) observed a correlation of heritability between traits for fruit and kernel and observed that the shape (width/length and thickness/length ratios) of the fruit and kernel had intermediate heritability of 0.46 and 0.53, respectively. The heritability of kernel traits was lower than that of shell traits (Martínez-García et al., 2019). The kernel/shell ratio is closely linked to shell hardness and thickness, and the inheritance of shell hardness is determined by individual genes, with the hard shell (low kernel/shell ratio) being dominant (Dicenta and García, 1993). The heritability of shell hardness (0.55) and thickness (0.51) had an intermediate value, and two quantitative trait loci (QTLs) were involved in shell hardness, with a major QTL located on Chr2 (Arús et al., 1999). In almonds, there were two kernel-weight loci (Kw-Q1 and Kw-Q2) in linkage groups 1 and 4 and two in-shell-weight loci (Shw-Q1 and Shw-Q2) in linkage groups1 and 2 (Sánchez-Pérez et al., 2007a). Fourteen putative QTLs controlling these physical traits were detected in the linkage map, corresponding to the six genomic regions of the eight almond linkage groups (Fernández i Martí et al., 2013). As mentioned above, the number of major and minor genes controlling the complex traits of stone shell and kernel remains unknown and little known about its genetics. Therefore, knowledge of these heritability values is of great significance for breeding kernel-using apricots. Although several important traits have been reported in similar crops, their underlying molecular mechanisms remain unclear.

Understanding the genetic mechanisms that control a specific trait would enable breeders to efficiently apply marker-assisted breeding through the development of DNA diagnostic tools and to consequently select seedlings with desired quality traits early in the selection process before the traits can be evaluated in the field. The link between genetic markers and a particular trait can be determined using genome-wide association studies (GWAS) based on next-generation high-throughput sequencing techniques. G WAS are widely used to dissect complex genetic traits (Cao et al., 2016; Fu et al., 2021). In the present study, we re-sequenced 339 apricot accessions and analyzed the diversity among the materials and the population structure of kernel-using apricots. We also analyzed the phenotypic variation and correlation between 19 traits of 222 accessions and calculated the broad-sense heritability of shell and kernel traits using variance of analysis (ANOVA). Finally, we used GWAS to further dissect the genetic foundations for their traits, and mine the candidate genes in the regions of the QTLs. The results of this study will provide more complete genetic information for these traits, and help improve the quality or yield in kernel-using apricot breeding.

## 2 Materials and methods

### 2.1 Plant material

A total of 339 apricot accessions were selected and analyzed to represent the entire range of phenotypic diversity and geographic distribution of apricots (**Supplementary Table S1**). In detail, the 312 cultivated apricot accessions were derived from North China (NC group, 114), Northwest China (NW group, 55), Central Asia (Xinjing [XJ] group, 17), Northeast China (NE group, 25), Europe (EU group, 23), South China (SC group, 24), kernel-using apricots (KU, 23), and one hybrid of *Prunus mume* (“Yanmei”). In addition, accessions of 27 individuals of wild apricots including 16 P*. armeniaca* L., seven *P. sibirica* L., and four *P. mandshurica* (Maxim.) Koehne (PsPma) were used. Of these, 180 accessions had been used in a previous study (Zhang et al., 2021). The trees of these accessions were planted at a density of 5 × 5 m in the Chinese National Germplasm Repository for Plums and Apricots at the Liaoning Institute of Pomology (Xiongyue, China). The orchards were subjected to conventional field management and pest control practices.

### 2.2 DNA extraction, library construction, and sequencing

The leaves of all accessions were sampled from the Chinese National Germplasm Repository for Plums and Apricots located at the Liaoning Institute of Pomology (Liaoning, China). Genomic DNA from 159 accessions was extracted from fresh young leaves using the modified cetyl trimethylammonium bromide method. Approximately 1 μg of genomic DNA for each accession was fragmented by sonication to a size of 300–500 bp with a Covaris S2 Focused Ultrasonicator (Thermo Fisher Scientific, Massachusetts, USA), and then end-polished, A-tailed, ligated with the full-length adapters, and polymerase chain reaction (PCR) amplified. A whole-genome shotgun paired-end library was prepared and sequenced on an Illumina HiSeq X Ten platform at BMK (Beijing, China) using the PE150 (paired-end, 125 bp) strategy according to the manufacturer’s recommendations. Raw reads were filtered prior to genome mapping. The adaptor sequences in the raw reads were removed, and ambiguous and low-quality bases from the start or end of the raw reads were trimmed using SOAPnuke (Chen et al., 2018). Of the 339 accessions used in this study, the raw reads of 180 accessions were obtained from the Sequence Read Archive of the National Center for Biotechnology Information (NCBI) (https://www.ncbi.nlm.nih.gov/sra/) with inquiry number PRJNA705053 (Zhang et al., 2021). The raw data for the other 159 accessions were deposited at NCBI under the BioProjet number PRJNA917328.

### 2.3 Genotyping and population structure analysis

The remaining high-quality paired-end reads were mapped to the apricot reference genome (Yinxiangbai v1.0) using BWA (version 0.7.15-r1140) with the command “mem –t 4 –k 32 -M” (Li and Durbin, 2009). To reduce mismatches generated by PCR amplification before sequencing, Picard Tools (version 2.23.6) were used to remove duplicate reads. Single-nucleotipde polymorphisms (SNPs) and insertions/deletions (InDels) were identified using SAMtools v1.4 (Li et al., 2009), and low-quality SNPs were filtered out with parameters for minimum minor allele frequency (MAF)< 0.01, missing data per site > 0.1, and individual depth > 4, and finally converted into a variant call format file (VCF). After alignment, SNPs/InDels were refiltered on a population scale using the Genome Analysis ToolKit version 4.1.2 (GATK4.1.2) (McKenna et al., 2010).

The nucleotide diversity (π), Watterson’s estimator (θ_W_), gene diversity/heterozygosity (*He*), *F_ST_*, and Tajima’s *D* for apricot populations were estimated using VCFtools (v0.1.14) (Danecek et al., 2011). For the window calculation, the window size was set to 20 kb with a step size of 10 kb across the apricot genome. Only the windows that comprised ≥ 8 kb effective covered region were considered. A distance matrix for all samples was calculated using genotype VCF files in VCF2Dis (https://github.com/BGI-shenzhen/VCF2Dis), and a phylogenetic neighbor-joining tree was constructed based on the distance matrix using the software PHYLIP v3.68 (http://evolution.genetics.washington.edu/phylip.html), and presented using MEGA7 (Lee et al., 2014). Principal component analysis (PCA) was performed to evaluate the genetic structure of the populations using the MingPCA software (V6.0.1) (Patterson et al., 2006). Population genetic structure was determined using ADMIXTURE (v1.3) (Alexander et al., 2009), which implements a block relaxation algorithm. To evaluate the best genetic clusters *K*, the cross-validation (CV) error was tested for *K* values between 2 and 10; *K* = 6 was chosen because the lowest CV error was observed at *K* = 6. For each sample, if the component for a sample within the major group was ≥ 0.60, the sample was classified as this major group, and the remaining samples were deemed admixed. Linkage disequilibrium (LD) was calculated using PopLDdecay v3.31 (Zhang et al., 2019) with parameter “-MAF 0.05 -Miss 0.1 -MaxDist 1000” in the entire investigated apricot samples and groups. The LD decay was calculated based on the squared correlation coefficient (*r* ^2^) values between the two SNPs and the physical distance between the two SNPs.

### 2.4 Phenotypic data

Phenotypic measurements were conducted during fruit ripening in 2019 and 2020. One hundred representative fruits were randomly collected from the outer canopy of the tree, and whole mature nuts were removed from the fully mature fruits, washed and dried for investigation. The shapes of the nuts and seed kernels were measured using Vernier calipers, including stone shell or kernel length (SL or KL), width (SW or KW), thickness (ST or KT), length/width ratio (SL/SW or KL/KW), and length/thickness ratio (SL/ST or KL/KT). Samples were randomly selected to obtain 30 records. The average values of dry shell weight with kernel (SDW) and dry kernel weight (KDW) of the samples were recorded using an electronic balance, and the kernel/shell ratio (KR) was calculated as (KDW × 100)/SDW. All samples were analyzed out with an average of 100 seeds.

The shell breaking force (SBF) and hardness (SH) of the nuts were measured using the TPA module of the TMS-PRO physical property analyzer (Food Technology Corporation, Virginia, USA), and 2500 N sensors (diameter 35 mm) was selected. The parameters were set as follows: downstream speed 2.0 mm s^-1^, tested speed 1.0 mm s^-1^, upstream speed 2.0 mm s^-1^, compression deformation of the sample as 30%, and the trigger force as 1.0 N. Twenty nuts were randomly selected for each accession. The nut shell thickness (STH) was measured using a Vernier caliper at the thinnest point in the middle of the cracked shell. The average STH per sample was recorded for the 20 nuts.

All the dry nuts of each sample were broken open and the seed kernel were removed and, then, the shell was crushed into a fine powder with a grinder. After filtering although a 60-mesh screen, the powder was stored in a plastic Ziplock bag to determine the acid insoluble lignin content. The acid insoluble lignin content (SLC) was determined using the Klason method (Alba et al., 2000), and each sample was evaluated thrice.

Kernel bitterness (SK) was evaluated in 2020. The nuts of mature fruit were cracked, and each sample was tested by two people, who classified its flavor as bitter (0) or non-bitter (sweet or to slightly bitter, 1). The value of pistil abortion (AR) is usually determined as the ration between the number of abnormal flowers (including the absence of pistils, withered ovaries, or abnormal styles) and the total number of flowers from the entire tree. More than 300 flowers from short shoots were randomly sampled around the canopy at the full-blooming stage.

The broad-sense heritability (*H^2^*) of these traits was calculated based on the components of variance between and within cultivars using one-way ANOVA as follows:


$$H^{2}=\frac{V_{1}-V_{2}}{V_{1}+(\text{r}-1)V_{2}}$$

where V_1_ is the inter-cultivars variance, including environmental variance and genotype variance; V_2_ is the environmental variance within the cultivar; and *r* is the number of replicates in the sample (Shen, 1997). Correlations between each pair of traits were calculated using the default statistical method in Origin2020 software (https://ea-origin.en.softonic.com). Correlation analysis of different traits was performed using the corrplot R package.

### 2.5 Genome-wide association study

A total of 3,472,166 SNPs were used in the GWAS. General linear models (GLMs) and generalized linear mixed model (GLMM) were generated using the Genomic Association and Prediction Integrated Tool (GAPIT 6.0 Version) (Lipka et al., 2012) and fastGWA-GLMM (version 1.94.0beta) (Jiang et al., 2021). The SNP data were converted to character, as described in the user manual, the population structure was the kinship matrix calculated using TASSEL 5.0, and the Q matrix (PCA = 3) was obtained from GAPIT 6.0. All GWAS parameters were set to default values. The significantly associated SNPs in the GLM method were determined by the critical threshold of –log_10_(*P*) ≥ 8.0 at *P* = 0.05 level [–log_10_(0.1/3.47 × 10^-6^) = 7.8], and the significance threshold in the GLMM method was set to 6.5. To reduce false positives and increase the statistical accuracy, the Bonferroni-corrected (FDR) *P*-value threshold was set at *P*< 0.01. We considered a QTL reliable when a QTL was consistently detected in two seasons or methods. These QTLs were named as “qtn” + trait name abbreviation + scaffold + detected QTL order on chromosome, such as *qtnSTH_4.1*.

### 2.6 Identification of candidate genes

Candidate gene analysis of the kernel taste trait was performed within haploblock regions. Considering that LD decay was approximately 20–50 kb for the different groups, the physical locations of QTLs on the reference genome were identified by mapping the 50-kb interval upstream and downstream sequences of the significant SNP to the genome. Genes within the QTL regions, together with their functional annotation information of the *Prunus armeniaca* “Yinxiangbai” genome (Zhang et al., 2021), were employed to identify putative candidate genes for each trait QTL.

## 3 Results

### 3.1 Re-sequencing and genetic variability

A total of 339 apricot accessions were selected in this study, of which 159 were re-sequenced using the Illumina platform and 180 were previously reported (Zhang et al., 2021). These re-sequenced accessions were representative of a Chinese core collection that covers the widest genome diversity. The geographic distributions of these accessions include NC, NW, XJ, NE, EU, SC (**Supplementary Table S1**). Notably, we selected 28 accessions from Hebei Province, where kernel-using apricot was first cultivated.

A total of 1.86 Tb clean data were generated with an average sequencing depth of 20.70X per individual after filtering out low-quality reads. The quality of the sequencing data was high with a Q30 > 92.6%, and the GC content ranged from 38.69 to 41.06%. The reads were mapped against the “ Yinxiangbai” genome to identify genomic variants, yielding ~82.87% coverage of the genome by at least five reads for each accession. The mapping rate ranged from 88.58 to 98.49%, with an average of 97.96% (**Supplementary Table S2**). The genomic variants were then detected using BWA software, obtaining a final set of 7,004,094 SNPs and 1,643,354 small InDels, with a density of 27.94 SNPs/Kb and 6.55 InDels/Kb on average (**Supplementary Figure S1**; **Supplementary Table S3**). Approximately 33.54% (2,355,512) of the SNPs were located in genic regions, and 10.51% (737,984) were in coding regions.

### 3.2 Population structure, linkage disequilibrium, and genetic diversity

**Figure 2:**
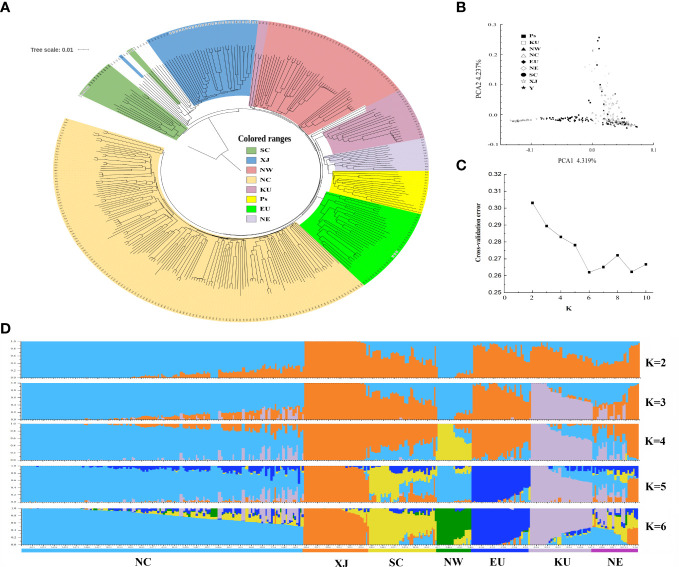
The population structure inferred based on genetic variations for apricot populations. **(A)** phylogenetic tree, **(B)** principal component analysis (PCA), **(C)** cross-validation error trend, and **(D)** population structure of 339 apricot accessions. Cultivated accessions include accessions from NW (Northwest of China), NC (North China), EU (Europe), NE (Northeast China), SC (South China), XJ (Xinjiang of China and Central Asia), KU (kernel-using apricot), and Ps (*P. sibirica* and *P. mandshurica*) accessions.

To investigate the population structures of these apricot accessions, we first constructed a rooted neighbor-joining (NJ) tree with *Prunus dasycarpa* (Ehrh.). Borkh was used as an outgroup based on these high-quality SNPs indentified (**Figure 2A**). The phylogenetic tree showed that 14 accessions from SC and Japan formed the first clade, and the ancestry of these accessions was mixed with that of the Japanese apricot (*P. mume*). The other accessions formed at the second clade, which included five main groups. The group I included most wild common apricots and all of accessions from XJ; group II contained majority accessions from NW and three kernel-using accessions (KU), and group III contained the majority KU cultivars, NE accessions and the close species (*P. sibirica* and *P. mandshurica*) of wild apricot; and group IV included cultivated apricots in the EU group; and group V consisted of all cultivars from NC, some NW cultivars, and mixed with several native wild common apricots. The result showed that most kernel-using accessions clustered with the NE accessions, *P. sibirica*, and *P. mandshurica*.

Next, we performed PCA of these samples (**Figure 2B**). The first two principal components (PCs) explained 8.56% of the total genetic variation, at 4.32 and 4.24% for PC1 and PC2, respectively. PC1 clearly discriminated accessions with distant kinship to the XJ cultivars, including accessions from wild common apricot, the EU/SC groups, and the NW/NC group. PC2 separated groups among NE, KU, and *P. sibirica*. However, groups could not clearly distinguish landraces between NW and NC based on their geographical distribution, which may be owing to frequent genetic exchanges generated by selection or introduction, reflecting the complex domestication and breeding history of cultivated apricots.

The population structure and genetic ancestry of the 339 accessions were inferred using ADMIXTURE, with K values ranging from 2 to 10 (**Figures 2C, D**). The most suitable number of ancestral populations was determined to be *K* = 6, at which the lowest cross-validation error of 0.262 was obtained (**Figure 2C**). The results of the population structure analysis recapitulated the clear-cut genetic differentiation in the phylogenetic tree and PCA. This resulted in six distinct population clusters: NC, XJ, SC, NW, EU, KU, and NE (**Figure 2D**). Notably, in each population, there were always several individuals of ancestry from other populations (**Supplementary Table S4**). For instance, in the KU population, R20, R22, R24, and R27 had backgrounds similar to those of the NC population.

To better understand the adaptive selection of kernel-using apricot populations, we explored the genetic diversity and divergence of different populations using nucleotide diversity (π), Watterson’s estimator (θ_W_), gene diversity/heterozygosity (He), and Tajima’s D. Across the genome, π, θ_W_, and HE for the KU population were estimated to be 6.19 × 10^−3^, 6.03 × 10^−3^, and 6.04 × 10^−3^, respectively, indicating that the level of diversity and heterozygosity of kernel-using apricot are high; however, there were a large number of medium frequency alleles in the population, which may be caused by the bottleneck effect of the population. The Siberian apricot (Ps) had the highest level of genetic diversity (π = 7.05 × 10^−3^ and θ_W_ = 7.46 × 10^−3^), whereas the cultivated group NC had the lowest level with π = 5.68 × 10^−3^ and θ_W_ = 5.80 × 10^−3^ (**Table 1**). Linkage disequilibrium analysis supported similar results, in which LD decayed with an increase in the physical distance between SNPs in all groups (**Supplementary Figure S2**). When the LD coefficient decreases to the baseline level, the corresponding fragment length is defined as the LD decay value of the corresponding crop. The results indicated that there were a few regions with long LD blocks in these populations. Different patterns of LD decay were observed in different groups. The KU group had the highest baseline LD coefficient (0.16) and physical distances at which the LD decayed (50 kb), whereas the EU and NC groups showed the longest distance (> 100 kb) and shortest distance (< 20 kb), respectively.

**Table 1:** Genetic diversity and Tajima’s D in apricot.

Group	NC*	KU	NEC	Ps	Total
Population size	38	21	11	10	339
**π** (10^-3^)	5.68	6.19	6.72	7.05	6.72
**θ**_W_ (10^-3^)	5.80	6.03	6.68	7.46	7.42
*H_E_* (10^-3^)	5.61	6.04	6.41	6.70	6.68
Tajima**’**s D	-0.0834	0.1018	0.0367	-0.2011	-0.2986

Diversity (1 × 10^−3^) is described by nucleotide diversity (π), Watterson’s estimator (θ_W_), and gene diversity/heterozygosity (He), and reported per bp. *NC here represents only 38 samples from Hebei and Beijing.

### 3.3 Phenotypic data

**Figure 3:**
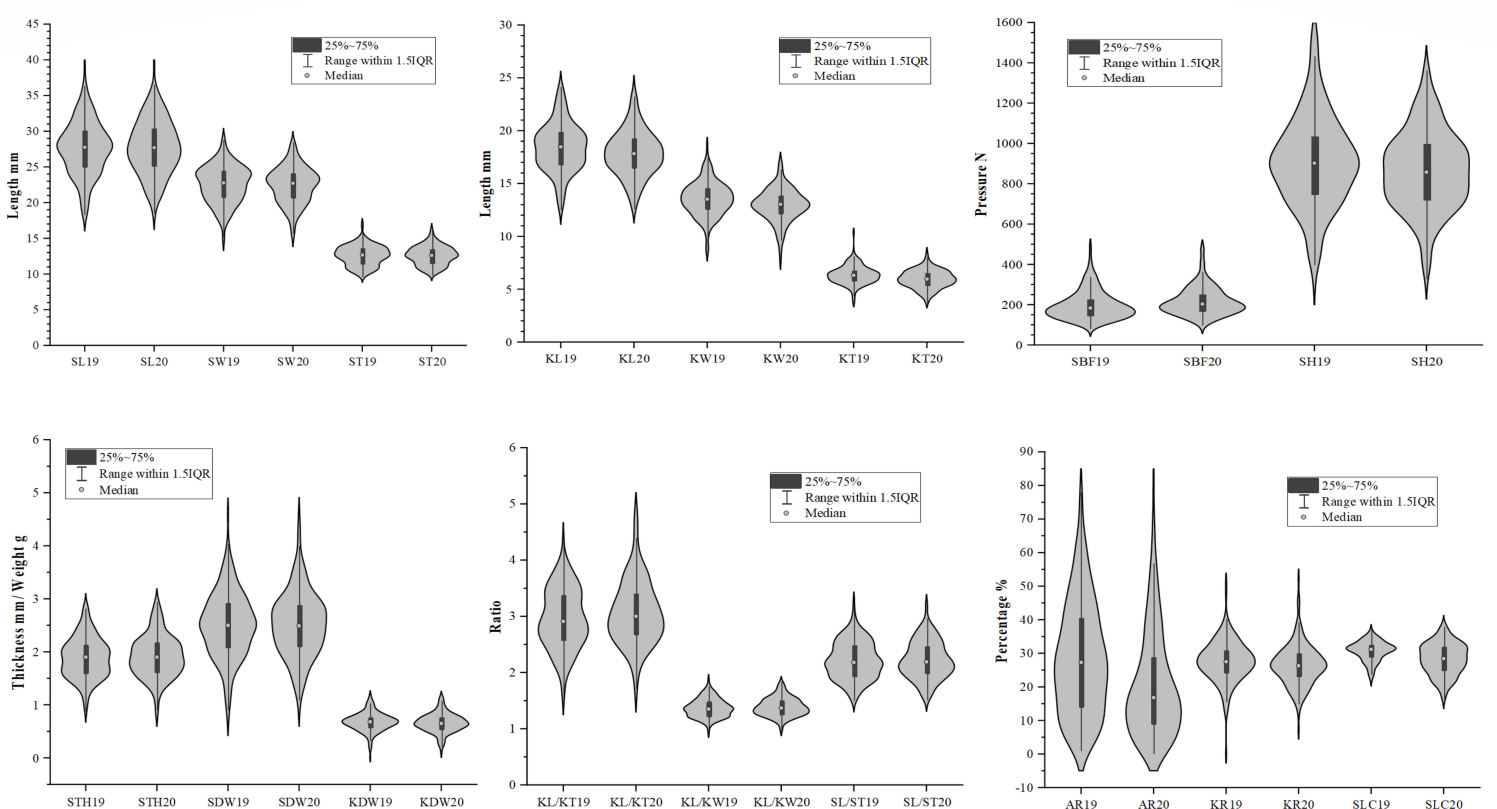
Violin plots diagrams of 18 traits of 222 apricot accessions in 2019 and 2020.

Two hundred and twenty-two accessions were evaluated for 19 different traits, including 10 kernel traits, seven seed traits, KR, and AR. The normal distribution and maximum, minimum, and mean values of these traits for different accessions are presented in **Figure 3** and **Supplementary Table S5**. A high degree of variation was found in important quantitative characteristics related to kernel traits. The highest SDW was recorded for 80D05 4.62 g, whereas the lowest SDW of 1.10 g, was found in Baihuanna with a coefficient of variation (CV) of 24.51%. The mean SDW was recorded as 2.52 ± 0.62 g. A total of 10 genotypes had SDW > 3.50 g. Regarding SL, values varied from 18.58 mm in Lve to 39.52 mm in Tianhuangkouwai, with a CV of 13.75%, whereas SW ranged from 15.18 mm in Zaojinmi to 28.81 mm in Zhanggongyuan with a CV of 11.48. The average SL and SW were 27.70 and 22.56 mm, respectively. Broad sense heritability (*H^2^*) was estimated for 13 traits (**Supplementary Table S5**). High average values of *H^2^* (> 85%) were observed for SL (94.46%), SW (88.96%), ST (86.84%), SL/SW (92.00%), and SL/ST (92.01%).

Because the hardness of the stone of the kernel-using apricot increases the cost of opening the shell and using the kernel, STH, SBF, SH, and SLC were also used for germplasm evaluation in this study. The variability in SH was very high in different years, and the CV was 24.89% in 2019 and 22.60% in 2020. Values for SH varied from 305.31 N in Lve to 1573.37 N in Taohexing, whereas STH ranged from 2.95 mm in Momoxing to 0.80 mm in Luren with a CV of 20.47%. Mean SH and STH values were 889.25 N and 1.90 mm, respectively. In this study, a total of 18 genotypes had STH< 600 N or STH< 1.40 g. The lowest SLC was recorded for Weixuan NO1 16.54%, whereas the highest SLC of 38.02%, was found in Wantianxing with a CV of 13.08%. The mean SLC was recorded as 28.23 ± 4.68%. Compared with STH (80.43%), the *H^2^* values of other shell-opening traits were lower in SH (61.10%) and SBF (17.08%).

The seed kernel is the main edible portion of kernel-using apricots and a higher KR ratio is an important agronomic attribute. The KR value ranged from 13.78% in Gaxing to 51.8%, in Luren with an average of 27.20% and CV of 21.83. A total of 11 accessions had KR > 35%. The KDW ranged from 0.17 g, in Tangwangchuan Dajiexing to 1.19 g in 80D05, with mean of 0.67 g and CV of 26.54. Thirteen genotypes had a KDW > 0.90 g. The distribution of KL ranged from 12.68 mm (in Caopixing) to 24.16 mm (in Tianhuangkouwai) with an average of 18.17 mm, whereas the distribution of KW ranged from 7.57 mm (Zaojinmi) to 18.58 mm (80D05) with an average of 13.28 mm. The CV of KL and KW were 12.32 and 11.82%, respectively. The KT ranged from 3.81 mm, in Liaoxing5 to 10.36 mm in Luren with an average of 6.34 mm and a CV of 13.52. The heritability of kernel traits was usually lower than that of stone shell traits. The *H^2^* of KL, KW, KT, KL/KW, and KL/KT were 89.92, 88.08, 69.31, 86.37, and 76.32%, respectively. The SK trait is an important quality index in kernel-using apricots. The kernel taste of 123 accessions was sweet and that of the remaining 99 was bitter.

**Figure 4:**
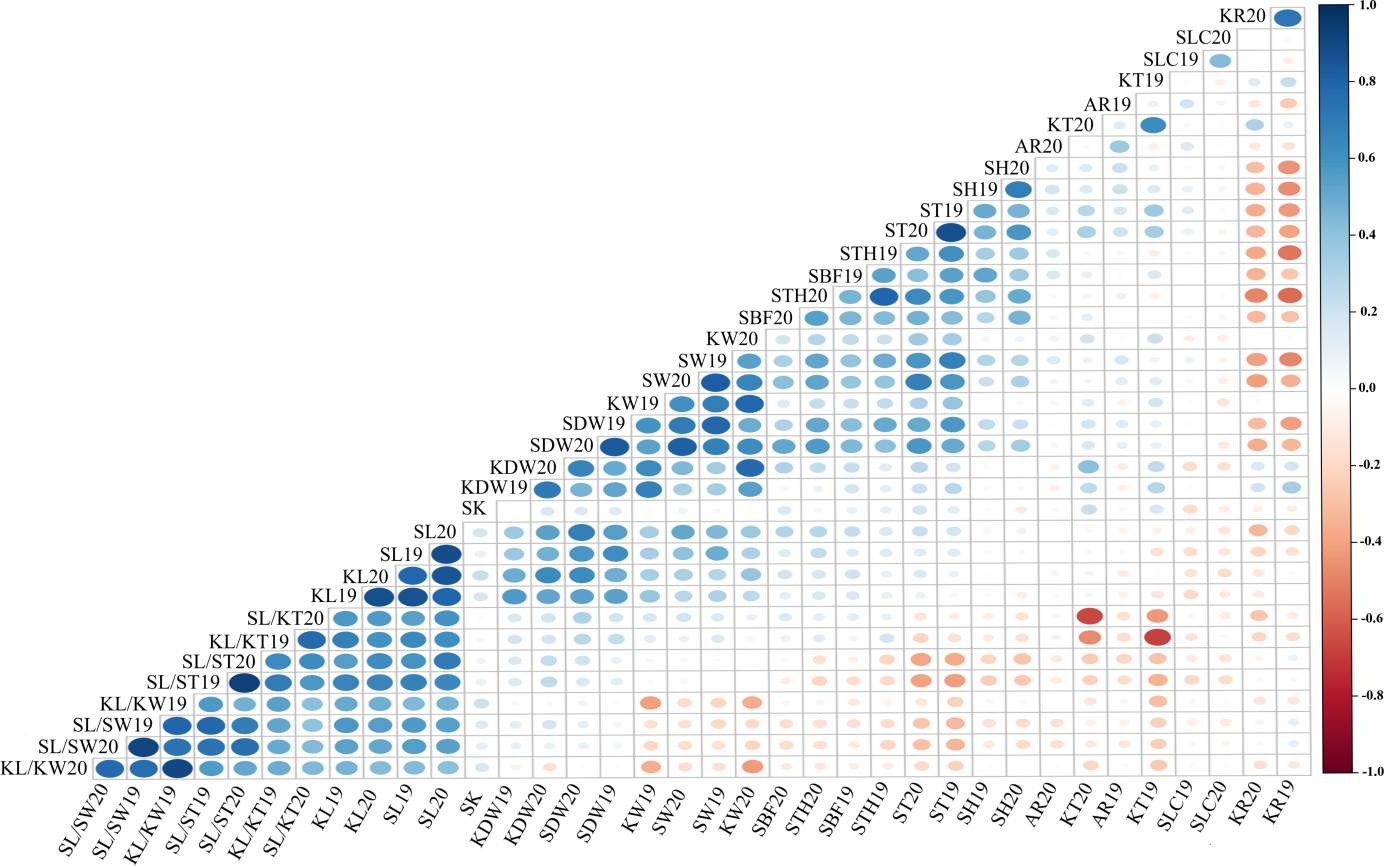
Correlation analysis of 18 traits in 2019 and 2020. The color and size of dots represent the correlation coefficient and *p*-value of corresponding traits respectively. Blue is positively correlated; red is negatively correlated.

The Pearson’s correlation coefficient is an important statistical method for quantifying the association or coherence between two variables. The data for all traits were at an extremely significant between 2019 and 2020, with correlation coefficients ranging from 0.475 (AR) to 0.906 (SL/ST). The Pearson correlation coefficient matrix for the 18 quantitative traits is shown in **Figure 4** and **Supplementary Table S6**. There was a highly significant correlation (*P*< 0.01) between the size of the stone (SDW, SL, SW, and FT) and kernel (KDW, KL, and KW). However, the KT correlations showed non-significant values for SL, SW, and KL. Highly significant correlations were also found between stone shape and kernel traits such as KL/KW, KL/KT, SL/SW, and SL/ST. The negative correlation between SH and SL/ST was −0.280, whereas it was 0.365, 0.334, 0.556, and 0.559 between SH and SDW, SH and SW, SH and ST, and SH and SBF, respectively. The correlation between STH and SH (0.493) was extremely significant (*P*< 0.01), whereas, STH also had a similar correlation with SH and other traits but no correlation between STH and SL/ST. Highly significant positive correlation values were observed between KR and KDW (0.395) and KT (0.400); however, negative correlations were observed between KR and STH (−0.579), and SH (−0.513), and stone sizes (SDW, SL, and KW). Of note, the correlation coefficient between KR and KL or KW was not significant, but that between KR and KT was significant. There was no reproducible correlation between SLC and other traits in different years, but a positive correlation was found between SLC and SH19 (0.231) in 2019.

### 3.4 Genome-wide association study and candidate genes prediction

**Figure 5:**
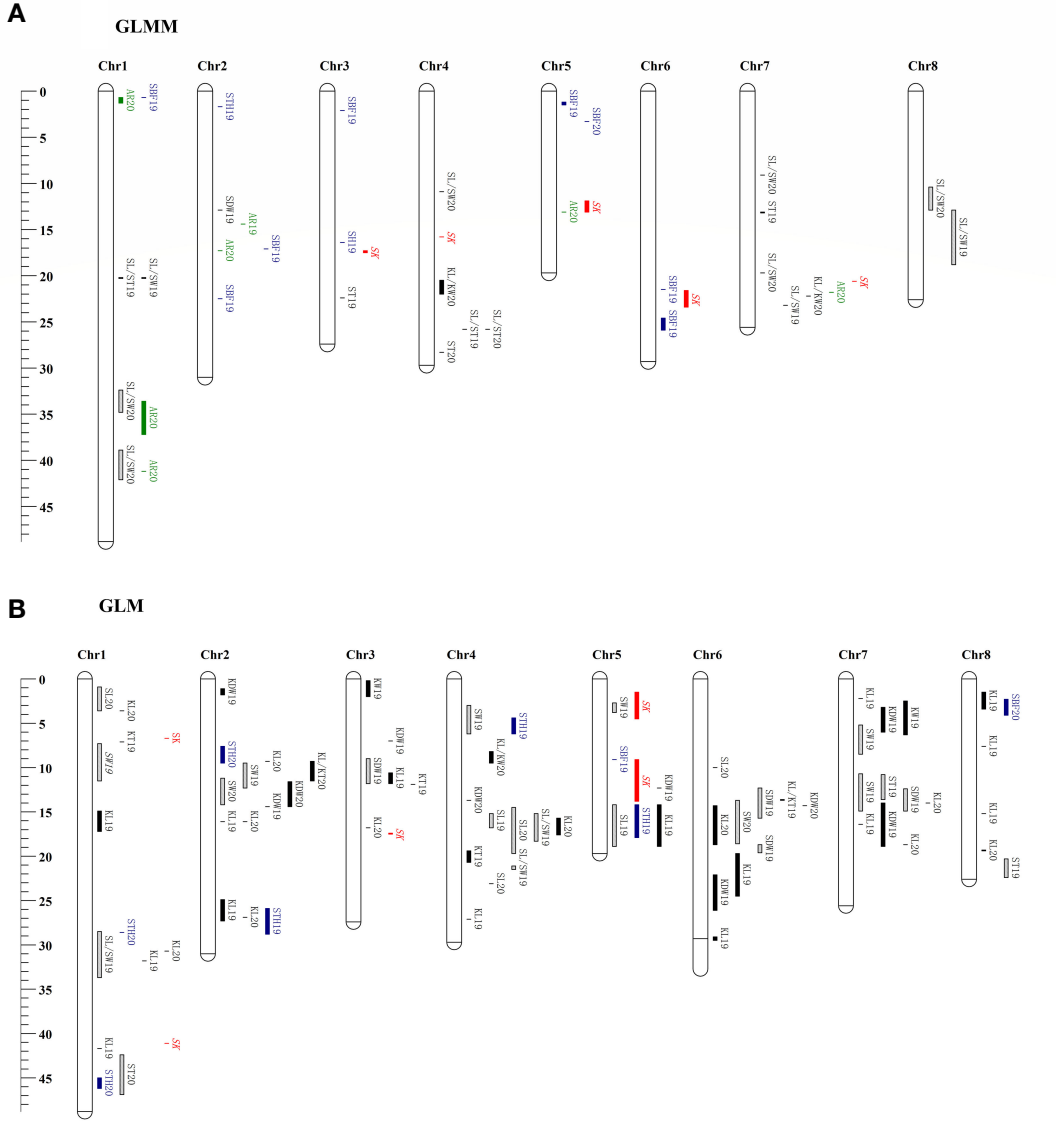
Chromosomes location of reliable QTNs detected using the GLMM **(A)** and GLM **(B)** method in this study. Genetic distance scale in physical position (Mbp) is placed at left margin. Green is for the QTNs of abortion rate (AR), red is for the QTNs of sweet kernel (SK), black is for the QTNs of kernel traits, cyan is for the QTNs of shell traits, and the box with a slash line is for the QTNs of stone traits.

The SNPs of 222 samples with phenotypic data were selected from 339 samples, and a phylogenetic matrix (**Supplementary Figure S3**) was constructed using the SNPs filter using MAF 0.05. The GWAS using the GLMM method revealed that 40 QTLs were associated with the10 traits investigated (**Figure 5A**), whereas no significant loci were detected for the remaining nine traits. All QTLs are list in **Supplementary Table S7**. The highest number of associated QTLs was observed on chromosome 1 (8) and the lowest in chromosome 8 (2). A total of 82 significant QTLs were found to be associated with the 14 traits in 2 years using the GLM method (**Figure 5B**), and no QTL was detected for the remaining five traits. The number of significant QTLs varied across various traits, ranging from 1 for KL/KW to 26 for KL (**Supplementary Table S7**). The chromosomal distribution of all identified QTLs revealed that Chr1 had the maximum number of significant QTLs, which were not evenly distributed in the genome, and four QTLs hotspots on chromosomes 2, 4, 6, and 7 were observed (**Figure 5B**). The percentage of phenotypic variation explained (PVE) and effect sizes for all the QTLs are shown in **Supplementary Table S7**. However, no significant associated loci were detected by either method for the two traits, such as AR and SLC.

Two repeatable QTLs, *qtnKL_2.2* and *qtnSL/ST_4.1*, were located on chromosomes Chr2 16,051,059–16,051,521 bp (PVE 21%) and Chr4 25,762,245 bp (PVE 7%), respectively. According to the recently released genome sequence of apricot (Zhang et al., 2021) and gene annotation information, 35 candidate genes for the traits of interest were detected around the two repeatable QTLs (**Supplementary Table S8**), and 23 were annotated. There were 12 candidate genes at the *qtnKL_2.2* locus, eight of which were annotated with the corresponding genes in the genome. These genes included, two interleukin-1 receptor-associated kinase [EC:2.7.11.1], 3-oxoacyl-[acyl-carrier protein] reductase [EC:1.1.1.100], DNA-directed RNA polymerase III subunit RPC8, two large subunit ribosomal protein L18Ae, leucine-rich PPR motif-containing protein, and alpha-amylase [EC:3.2.1.1]. Among *qtnSL/ST_4.1*, candidate genes were related to cell division and hormone synthesis, for example, cytochrome C oxidase assembly factor 1, pyruvate kinase [EC:2.7.1.40], cell division cycle 20-like protein, chitinase [EC:3.2.1.14], ATP-dependent RNA helicase UAP56/SUB2 [EC:3.6.4.13], somatic embryogenesis receptor kinase 1 [EC:2.7.10.1 2.7.11.1], DNA-directed RNA polymerase III subunit RPC2 [EC:2.7.7.6], and zinc finger SWIM domain-containing protein 3.

Nine reliable QTL that overlapped or clustered in Chr1 30,697,092–31,791,632 bp for KL, Chr1 32,42–33,70 Mb for SL/SW, Chr2 11.16–12.32 Mb for SW, Chr2 14.35 Mb for KDW, Chr2 26.88–27.25 Mb for KL, Chr4 14.54–15.17 Mb for SL, Chr5 1.16–3.30 Mb for SBF, Chr7 13.99–18.74 Mb for KL, and Chr8 12.88 Mb for SL/SW were identified in at least two years or methods, and these QTLs explained 6.50–19.76% of total phenotypic variation, respectively (**Supplementary Tables S7**, **S8**). Candidate genes and annotations of reliable QTLs (or clusters) are summarized in **Supplementary Table S8**. On Chr1, the QTL region of the SL/SW was detected in genes associated with cell division, DNA replication, and endocarp development, such as the replication licensing factor MCM2 [EC:3.6.4.12], DNA gyrase subunit B [EC:5.99.1.3], xyloglucan galactosyltransferase MUR3 [EC:2.4.1.-], shikimate O-hydroxycinnamoyltransferase [EC:2.3.1.133], beta-fructofuranosidase [EC:3.2.1.26], biotin synthase [EC:2.8.1.6], and cyclin-dependent kinase 12/13 [EC:2.7.11.22 2.7.11.23]. The QTL segment of SW in Chr2 was annotated to cell growth regulation genes, such as the transcription factor MYB, MADS-box transcription factor, gibberellin 20-oxidase [EC:1.14.11.12], gibberellin receptor GID1, proliferating cell nuclear antigen, fructokinase [EC:2.7.1.4], protein glucosyltransferase [EC:2.4.1.-], and (+)-neomenthol dehydrogenase [EC:1.1.1.208]. Furthermore, SNPs clustered in regions associated with more than one trait (SW, SL, KL, SDW, and KDW) and were identified on chromosomes 2, 4, 6, and 7.

### 3.5 Analysis of significant single-nucleotipde polymorphisms associated with sweet kernel trait

**Figure 6:**
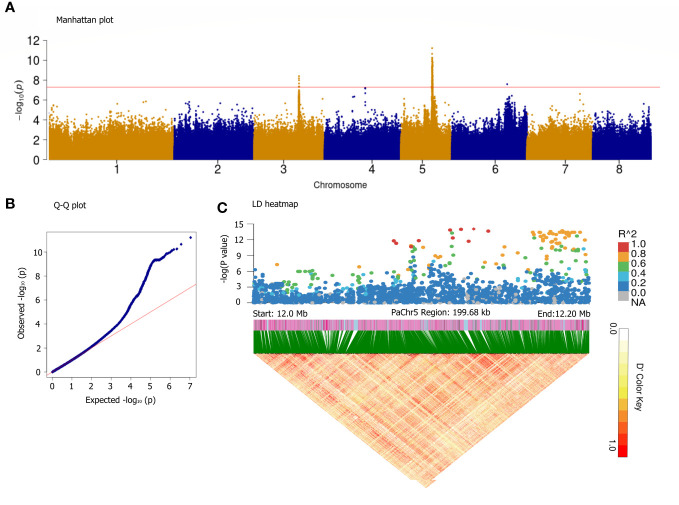
GWAS for sweet kernel trait base on the GLMM method. Manhattan plot **(A)**, Q-Q plot **(B)**, and LD heatmap in the 12.0–12.2 Mb segment on the Chr5 **(C)**. The x-axis represents the position along a chromosome, the y-axis represents –log_10_ (*p*-value), and the red line indicates significance threshold at [–log_10_(0.1/3.47*10^−6^) = 7.8]. A representation of pairwise *r* ^2^ values (a measure of LD) among all polymorphic loci in the LD heatmap.

It is generally believed that almond sweetness is a quality trait; however, the number of associated regulatory genes remains uncertain (Negri et al., 2008). Interestingly, the GWAS using GLMM (**Figures 6A, B**) and GLM (**Supplementary Figure S4**) models for the SK detected five significant peaks on all chromosomes. Only two reliable loci assigned to the Chr3 (17.34–17.51 Mb) and Chr5 (11.88–13.80 Mb) were significantly associated with SK (*P*< 10^−8^). The location of SK on Chr5 is similar to that reported in almond, indicating that they were a homologous gene controlling SK trait (Sánchez-Pérez et al., 2019). Most of the variants with the highest peak association were assigned to approximately 12,131,145 bp on Chr5; therefore, a haplotype block spanning 12.0–12.2 Mb on Chr5 is also shown in **Figure 6C**. On Chr5, 322 candidate genes were predicted within the haploblock regions of significantly associated loci (**Supplementary Table S9**). In the six of these genes, the transcription factor MYC2 corresponded to the previously reported genes, whereas 18 candidate genes were new, and their functions were derived from the annotated information in near 12.12 Mb, such as methionine-gamma-lyase [EC:4.4.1.11], glyoxal/methylglyoxal oxidase [EC:1.2.3.15], cytochrome P450 family 78 subfamily A, cardiolipin-specific phospholipase [EC:3.1.1.-], acetylajmaline esterase [EC:3.1.1.80], and endoglucanase [EC:3.2.1.4].

There were 13 annotated genes on the 17.34–17.51 Mb segment of Chr3 (**Supplementary Table S9**). Among them, three genes were related to plant transcription factor and encoded the MADS-box transcription factor, transcriptional elongation factor elongin-A, and general transcription factor 3C. *Pa03g10859.1*, *Pa03g10860.1*, *Pa03g10863.1*, and *Pa03g10871.1* were related to secondary metabolism and glycoside transport, such as citrate synthase [EC:2.3.3.1], V-type H+-transporting ATPase subunit D, actin related protein 2/3 complex, and galacturonosyltransferase 12/13/14/15 [EC:2.4.1.-]. The remaining six genes were considered responsible for encoding serine/threonine-protein phosphatase 5 [EC:3.1.3.16], ATP-dependent DNA helicase PIF1 [EC:3.6.4.12], aspartyl-tRNA(Asn)/glutamyl-tRNA(Gln) amidotransferase subunit A [EC:6.3.5.6 6.3.5.7], and 3 interleukin-1 receptor-associated kinase 4 [EC:2.7.11.1].

## 4 Discussion

### 4.1 Re-sequencing confirmed that most kernel-using apricots originated from the cross between the common and Siberian apricots

Owing to an unreliable cultivation history, the origin and taxonomic status of kernel-using apricot have been unclear. In recent years, these uncertainties have been addressed using molecular markers. The results of simple-sequence repeat markers suggested that *P. cathayana* is a natural interspecific hybrid between common apricot (*P. armeniaca*) and Siberian apricot (*P. sibirica*) (Zhang et al., 2014; Fu et al., 2016). In the present study, cluster analysis showed that most kernel-using apricots, which were actually *P. cathayana*, were derived from natural hybrids, whereas a few cultivars that did not have typical Siberian apricot characteristics were directly derived from common apricots. Based on maternal genetic characteristics, using chloroplast simple-sequence repeat markers, Zhang et al. (2018) further concluded that *P. cathayana* was formed by natural crosses between *P. armeniaca* and *P. sibirica*. Based on the resequencing data, functional enrichment of the different genes was conducted between the KU vs. NC, or KU vs. Ps groups, and these genes were significantly enriched in the carbohydrate metabolism pathway and the terpenoid and polyketide metabolism pathway (Zhang et al., 2021). Our results reconfirmed that the majority of the KU population (*P. cathayana*) was derived from natural hybrid; however, the genetic variation in KU was lower than that of the other groups because of their reproduction from a few genotypes by asexual grafting. Therefore, it is necessary to cross the common or Siberian apricots to expand the genetic background of kernel-using apricot.

### 4.2 Phenotypic evaluation facilitated trait improvement and selection in kernel-using apricot breeding

Studying trait variation in different cultivars is an important pre-requisite for breeding. The present study revealed a high level of variability in stone shell and kernel traits among apricot cultivars compared with previous study (Wani et al., 2017). All traits were highly polymorphic in all apricot accessions. SDW and KDW are the two most important indices of yield in kernel-using apricots, and KDW is a distinct property of kernel quality that distinguishes modern cultivars from their small-fruit wild ancestors. In this study, KDW showed a large variation from 0.17 g (accession Tangwangchuan Dajiexing) and 1.19 g (“80D05”), and SDW ranged from 0.72 to 4.62 g. In general, the KDW of the kernel-using apricot was the highest, followed by that of the fresh-using apricot, whereas that of the Siberian apricot was the lowest. Our results show that some varieties of fresh apricot cultivars with KDW greater than 1 g, such as Momoxing and Huangkouwai, could be used as potential parents for genetic improvement in kernel-using apricot breeding.

The physical traits of apricot shells do not affect the organoleptic characteristics of the kernel; nevertheless, they are very important properties in all harvesting and industrial processes and must also be considered during cultivar evaluation (Sánchez-Pérez et al., 2019). Shell traits included STH, SBF, SH, and SLC. In the present study, coefficients of variation of these traits ranged from 9.59 to 36.10%, indicating that these accessions had abundant genetic diversity. For example, Keziaqia, Saimaiti, Hacihaliloglu, Luren, Dashanxing, C202-1, and B110-2 are important parents for improving the kernel yield and shell processing breaking force properties.

The close relationship between traits could facilitate or hinder the breeding process, as the selection for a given trait could favor the presence of another desirable or undesirable characteristic of the fruit tree. In the present study, highly significant correlations were found between shell and kernel traits, which indicated that the endocarp and kernel have similar shapes, because the stone shell determines the final shape of the kernel that will be developed inside. Positive correlations were observed among the variables related to stone shell or kernel traits, except for KT. The correlation between SL and SW or ST was 0.525 and 0.202, respectively. The correlation between KL and KW was significant at 0.412, whereas that between KL and KT was insignificant. Regarding kernel yield, the positive correlation obtained between KR and kernel weight was highly significant, whereas a negative correlation was found between KR and stone weight or size. Moreover, KR inversely correlated with STH, SH, and SBF. Correlation coefficients in apricots showed that KR mainly depends not on KL and KW, but on KT (*r* ^2 = ^0.36). In almond, the in-shell/kernel ratio was positively correlated with shell weight (−0.82) and shell hardness (−0.84) (Sánchez-Pérez et al., 2007b). Our result is similar to that of almonds (Dicenta and García, 1993; Sánchez-Pérez et al., 2007b), and an important conclusion was that although the thickness and hardness of the shells do not affect the weight of the kernel, the final yield of the orchard is affected. Therefore, harvesting depends on the thickness of the produced kernels and their weight, which is independent of the shape of the shell or kernel. However, the correlation coefficient between KR and KT was extremely significant, suggesting that improvement in the KT trait would be beneficial for increasing kernel yield in breeding.

Apricot stone and kernel traits should be considered in kernel-using apricot breeding programs. The continuous variation exhibited by the quantitative traits that plant breeders deal with includes heritable and non-heritable components. The parameters of stone and kernel traits, including size, shape, and thickness, are heritable and must be considered in the design of crosses for breeding programs. Therefore, in the present study, the heritability of stone and kernel traits was determined based on a one-way ANOVA of apricots. The result showed that the heritability of the shell size and shape was higher than that of the kernel, and the heritability of SL was the highest (94.46%). High heritability estimates indicate that the selection of these traits is effective and less influenced by environmental factors (Shen, 1997). High heritability estimates were obtained for stone and kernel size traits, indicating that selection for these traits would be more effective. The heritability of KT was moderate (69.31%); however, it was highly correlated with kernel yield.

Although physical traits do not affect the organoleptic characteristics of apricot kernels, they are important in the processing industry. Stone shell traits include shell thinness and hardness and affect nut cracking. The heritability of STH, SH, and SBF was 80.43, 61.10, and 17.08%, respectively, which indicated that SH and SBF were highly affected by the environment during the process of shell opening. These results agree with the findings of the almond study (Socias i Company et al., 2009).

### 4.3 Genome-wide association study analysis identified quantitative trait loci and screened candidate genes for certain traits of the stone shell or kernel

Owing to differences in recombination rates, selective pressures, and effective population sizes, LD decay may vary across populations; for example, narrow-based germplasm groups have longer LD blocks than broad-based germplasm groups. In the present study, the LD decay in KU (~40 kb) was longer than that in the other studies, perhaps owing to a consistent cultivation environment that resulted in a narrow genetic background and vegetative propagation (Cao et al., 2014). The LD level was higher for domesticated (including XJ and EU) apricot than for the semi-wild group (NE), an observation that has also been reported in peach, soybean, and rice. Reported values include those for wild peach (~10 kb) (Tan et al., 2021) and improved variety (540–1640 kb) (da Silva Linge et al., 2021), wild soybean (~75 kb) and cultivated soybean (~ 150 kb) (Lam et al., 2010), wild rice (~ 10 kb) and cultivated rice (65–200 kb) (Xu et al., 2012). In the present study, we detected the QTLs of 19 traits using GWAS in apricots, and only some significantly associated loci or traits were detected in a certain year, which may be related to the rapid LD decay in apricot populations. We found that the LD values to the baseline for the entire population were ~20 kb, which is comparable with those estimated for the native Chinese plum (Huang et al., 2021) and peach (Cao et al., 2016).

The inheritance of kernel taste in almonds is characterized as a monogenic trait (Dicenta and García, 1993) and mapped to the central region of Chr5 (Dirlewanger et al., 2004). Subsequently, the gene underlying the SK locus was shown to be a transcription factor (bHLH2), which is considered to be responsible for regulating the transcription and expression of the P450 monooxygenase–encoding genes, involved in the amygdalin biosynthetic pathway (Sánchez-Pérez et al., 2019). Bitterness is determined by the presence of prunasin/amygdalin, which is synthesized in the bark and leaves of trees and then transported to developing kernels. In a previous study, data from spectrophotometric assays indicated that seed cyanoglucoside content cannot be regarded as a quantitative trait. The results of several F1 segregation populations suggested that all segregation ratios of SK can be explained by an inheritance mechanism based on five non-linked genes involved in two distinct biochemical pathways (Negri et al., 2008). Therefore, these genes control cyanoglucosid biosynthesis and transport in apricots. In the present study, we also found a 46.77 kb gene cluster encoding six MYC2 transcription factors, which are responsible for regulating amygdalin biosynthesis. Similar to the multi-gene hypothesis, we also detected a new reliable genetic locus of SK on the region of Chr3 in 17.34–17.51 Mb, which are related to glycoside transport, such as V-type H+–transporting ATPase and galacturonosyltransferase.

Kernel size is an important phenotypic trait that distinguishes kernel-using cultivars from fleshy cultivars. Discovering the major loci underlying the genes that determine stone and kernel size has been a high priority because of the importance of kernel size for growers’ profitability and requirement of breeders. Fruit size is a classic quantitative trait controlled by many loci. In almonds, a total of 14 putative QTLs controlling these traits were detected, including three for KW, three for KT, and four for KL, but the low phenotypic variance explained less than 30% of the variation found for all these traits (Fernández i Martí et al., 2013). In the current study, four repeatable QTLs for KL were identified on Chr1, 2, and 7, and one QTL each for SW, KDW, and SL was located on Chr2, 2, and 4, respectively. Notably, in the present study, many loci regulating stone or kernel size were trait-rich clusters on Chr1, 2, 3, 4, 6, and 7, although most loci could not be repeatedly detected in different years or using different methods. The results were similar for fruit or kernel size in other species, such as peaches (Cao et al., 2016), cherries (De Franceschi et al., 2013), apricots (Zhang et al., 2022b), and almonds (Fernández i Martí et al., 2013). Two QTLs for fruit size were mapped to Chr2 and Chr6 in the sweet cherry genome; one gene, PavCNR12, contributed to an increase in fruit size by increasing cell number, and the other gene was a member of the Cytochrome P450 (CYP) subfamily (PaCYP78A979), which is also a candidate gene for a fruit weight QTL identified in peaches (De Franceschi et al., 2013). In peaches, two genes encoding E3 ubiquitin protein ligase were mapped on Chr4 at approximately 2 Mb and on Chr5 at 8 Mb, and they could alter the number of cells and contribute to fruit size and weight (Cao et al., 2016). These candidate genes were also annotated on the QTLs in the present study, suggesting that these QTLs are co-located across species.

In addition, the QTLs for nut shape were located in Chr1, 5, and 7 bases on the linkage map with 56 simple-sequence repeat markers in almond (Fernández i Martí et al., 2013). However, in the present study, three reliable QTLs for stone-shape traits (*qtnSL/SW_1.2*, *qtnSL/ST_4.1*, and *qtnSL/SW_8.1*) were located on Chr1, 4 and 8, respectively. These SL/SW QTL regions were annotated as candidate genes associated with cell division, DNA replication, and endocarp development.

The structure of the apricot shells is important for industrial processing. In almonds, shell hardness is usually measured using the kernel percentage, which is the proportion of the dry kernel weight to the total stone weight (kernel and shell) and has been considered as a qualitative characteristic determined by a single gene (D/d) located in the middle of Chr2 (Arús et al., 1999). Dicenta and García (1993) considered shell hardness as quantitative, with medium heritability (56%). When the progeny were examined for quantitative traits, two QTLs were detected for shell hardness, a major QTL located in the same position as Chr2, and a minor QTL located in the distal part of Chr8 (Arús et al., 1999). In the present study, STH, SH, and SBF were used to evaluate the physical properties of the stone shell, and their heritability was 80.43, 61.10, and 17.08%, respectively. In addition, eight loci for STH and one locus for SH were unevenly located on Chr1, 2, 3, 4, and 5; however, these loci were not reproducible. QTLs for SBF were also found in Chr2, 5, and 8. We found locus *qtnSTH_2.2* on Chr2 in the region of 7.59–9.45 Mb, which is related to multiple tandem genes associated with lignin synthesis and regulation, such as phenylalanine ammonia-lyase (*Pa02g07108.1* and *Pa02g07109.1*), tyramine N-feruloyltransferase (*Pa02g07115.1* and *Pa02g07116.1*), three caffeic acid 3-O-methyltransferase genes (*Pa02g07215.1*, *Pa02g07216.1*, and *Pa02g07217.1*), and seven 3-hydroxyisobutyryl-CoA hydrolase genes. This result is consistent with the differences in expression at the transcriptional and metabolic levels (Zhang et al., 2022a). We still need to dissect trait-rich QTL regions and narrow QTL regions to reduce the number of candidate genes, and the development of marker-assisted selection tools with high accuracy and efficiency is necessary for kernel-using apricot breeding.

In apricots, in a perfect flower, the pistil is stronger and longer than the stamens and can successfully bear fruit after pollination. However, flower development in apricots is a complex process that is strongly affected by genetic factors and environmental conditions, which may result in pistil abortion. Pistil-aborted flowers can fail to bear fruit. Based on the linkage map of specific-locus amplified fragment markers, several QTLs of pistil abortion were detected and mapped to the middle regions of LG5 (equivalent to the current Chr2) and LG6 (equivalent to the current Chr1), with nine markers closely linked to it (Zhang et al., 2019). In the present study, we analyzed the genetic loci of AR by GWAS, and detected seven QTLs by the GLMM method, and only two loci (*qtnAR_2.1* in AR19 and *qtnAR_2.2* in AR20) were located on the Chr2 at 14.43–17.34 Mb, which was annotated to calcium-binding protein CML (*Pa02g08051.1*), transcription factor MYC2 (*Pa02g08065.1*), cell division protease (*Pa02g08066.1*), and glucuronyl/N-acetylglucosaminyl transferase (*Pa02g08067.1*) genes. This difference may have been caused by the different evaluation methods or detection accuracies.

There are two frequently QTL mapping methods for complex traits at present: one is genetic linkage analysis based on segregating populations derived from two parental lines, and the other is GWAS using natural variation population. For a specific locus, genetic linkage analysis can only detect polymorphic loci in the segregation population and two alleles segregated from each locus in a diploid species (Ruiz et al., 2010; García-Gómez et al., 2019), while GWAS can detect the genetic effects of multiple alleles (da Silva Linge et al., 2021; Fu et al., 2021). When multiple QTL control a trait, their alleles of positive or negative effect (increasing or decreasing trait value) tend to be dispersed among genetic stocks, with positive alleles at one or some loci but negative alleles at others (Xu, 2010). Therefore, allele dispersion or association phenomena appears in QTL mapping through WGAS approach (Xu, 2010), which may be the reason for the generally low PVEs of the alleles in this study. However, these important QTLs and candidate genes will be analyzed by quantitative real-time PCR or other methods during the critical development stage.

In the present study, we demonstrated the origin and genetic diversity of kernel-using apricots based on re-sequencing data. Seventeen physical traits of stone shells and kernels were studied for the first time in apricots by observing their quantitative nature, correlations, and heritability. Our results also identified QTLs and candidate genes associated with these physical parameters, kernel taste, and pistil abortion traits to provide a genetic framework for use in breeding program to improve the quality of kernel-using apricots.

## Data availability statement

The datasets presented in this study can be found in online repositories. The names of the repository/repositories and accession number(s) can be found in the article/**Supplementary Material**.

## Author contributions

QZ designed the research, conducted the data analyses, and wrote the manuscript; YPZ, NL, XM and CL collected the phenotyping data; WL made suggestions on methods and design; MX, SL, and YJZ participated in the management of test materials. All authors have read and agreed to the published version of the manuscript.

## Supplementary material

Supplementary Figure 1 The distribution of markers generated from the whole-genome resequencing of the 339 accessions. The mutation sites were scanned using overlap-free sliding windows, where the default windows length is 100 kb. (A) single-nucleotide polymorphisms (SNPs); (**B**) insertion/deletion (INDELs).

Supplementary Figure 2 The chart of linkage disequilibrium (LD) decay in different populations.

Supplementary Figure 3 Hierarchical cluster analysis and the kinship matrix in 222 apricot accessions. Red indicates high level, whereas low values are shown in green.

Supplementary Figure 4 GWAS for sweet kernel trait base on the GLM method. Manhattan plot **(A)**, Q-Q plot **(B)**.
